# Readmission after discharge from acute mental healthcare among 231 988 people in England: cohort study exploring predictors of readmission including availability of acute day units in local areas

**DOI:** 10.1192/bjo.2021.961

**Published:** 2021-07-19

**Authors:** David P. J. Osborn, Graziella Favarato, Danielle Lamb, Terri Harper, Sonia Johnson, Brynmor Lloyd-Evans, Louise Marston, Vanessa Pinfold, Deb Smith, James B. Kirkbride, Scott Weich

**Affiliations:** Division of Psychiatry, University College London, and Camden and Islington NHS Foundation Trust, UK; Division of Psychiatry, University College London, UK; Division of Psychiatry, University College London, UK; Department of Primary Care and Population Health, University College London, UK; Division of Psychiatry, University College London, and Camden and Islington NHS Foundation Trust, UK; Division of Psychiatry, University College London, UK; Department of Primary Care and Population Health, University College London, UK; McPin Foundation, London, UK; McPin Foundation, London, UK; Division of Psychiatry, University College London, UK; Mental Health Research Unit, Sheffield University, UK.

**Keywords:** In-patient treatment, social deprivation, psychotic disorders, epidemiology, community mental health teams

## Abstract

**Background:**

In the UK, acute mental healthcare is provided by in-patient wards and crisis resolution teams. Readmission to acute care following discharge is common. Acute day units (ADUs) are also provided in some areas.

**Aims:**

To assess predictors of readmission to acute mental healthcare following discharge in England, including availability of ADUs.

**Method:**

We enrolled a national cohort of adults discharged from acute mental healthcare in the English National Health Service (NHS) between 2013 and 2015, determined the risk of readmission to either in-patient or crisis teams, and used multivariable, multilevel logistic models to evaluate predictors of readmission.

**Results:**

Of a total of 231 998 eligible individuals discharged from acute mental healthcare, 49 547 (21.4%) were readmitted within 6 months, with a median time to readmission of 34 days (interquartile range 10–88 days). Most variation in readmission (98%) was attributable to individual patient-level rather than provider (trust)-level effects (2.0%). Risk of readmission was not associated with local availability of ADUs (adjusted odds ratio 0.96, 95% CI 0.80–1.15). Statistically significant elevated risks were identified for participants who were female, older, single, from Black or mixed ethnic groups, or from more deprived areas. Clinical predictors included shorter index admission, psychosis and being an in-patient at baseline.

**Conclusions:**

Relapse and readmission to acute mental healthcare are common following discharge and occur early. Readmission was not influenced significantly by trust-level variables including availability of ADUs. More support for relapse prevention and symptom management may be required following discharge from acute mental healthcare.

In the English National Health Service (NHS), acute mental healthcare is provided by crisis resolution teams (CRTs) and in-patient units. CRTs are specialist mental health teams providing short-term, intensive home treatment on a daily basis.^1^ They are offered by almost all NHS mental health providers in England (known as ‘trusts’) and are based on a care model in which multidisciplinary teams provide acute assessment, treatment and psychosocial support to patients in community settings. Readmissions for acute treatment after discharge from an index episode are common. For example, one study in two inner London trusts found that among almost 18 000 people discharged from CRTs, over 50% were readmitted for acute treatment within 1 year.^2^ Recently, there has been a focus on reducing acute readmission and relapse rates by prioritising interventions which offer preventive community mental healthcare.

The current study is part of a research programme regarding acute mental healthcare in England and acute day units (ADUs) (Acute Day Units as Crisis Alternatives to Residential Care (AD-CARE), NIHR ref. HS&DR 15/24/17). ADUs offer an alternative to in-patient admission and were previously referred to as day hospitals. They are currently available in around one-third of NHS trust catchment areas and typically offer people daily, multidisciplinary contact in the community for 6–8 weeks, providing a variety of interventions including peer support, groups, psychological work and pharmacological interventions.^3^ There is some older evidence that these units might reduce in-patient admissions,^4^ although a systematic review comparing ADU care with in-patient care found no difference in readmission rates.^5^ Using national administrative data for England, this study aimed to determine the proportion of people readmitted to acute mental healthcare after they had been discharged following an episode of acute psychiatric treatment. We also aimed to assess whether risk of readmission varied between NHS provider trusts with and without access to ADUs, and to determine the clinical and sociodemographic characteristics associated with readmission.

## Method

### Design and setting

We developed a cohort study to analyse readmissions to the acute care pathway following discharge after an index episode of acute mental healthcare in the NHS in England. We used administrative mental health services data from April 2013 to May 2015. The cohort was constructed from the Mental Health Minimum Dataset (MHMDS). This data-set comprises pseudo-anonymised, individual-level data from all secondary adult mental health services in England. Data are collated and released by NHS Digital. The data are derived from returns from NHS mental health providers (trusts). Annual data-sets include information about all in-patient, day treatment, out-patient and community-based secondary mental healthcare. They also include patients’ demographic characteristics. We combined annual data-sets to form a continuous cohort over the study period. We report our findings according to the RECORD (Reporting of studies Conducted using Observational Routinely collected Data) guidelines.^6^

### Participants

We included individuals discharged from NHS acute mental healthcare. This was defined as an episode of care provided by CRTs and/or in-patient admission to a psychiatric ward, between 1 April 2013 and 30 May 2015. Cohort entry was the date of discharge from an episode of psychiatric acute care (either from a CRT, in-patient ward or both); this episode is referred to as the *index* admission. Cohort exit was the first date of (a) readmission to acute mental healthcare (defined as a new episode of care provided by a CRT team and/or in-patient admission to a psychiatric ward), or (b) the end of the 6 month follow-up period following index admission discharge.

We excluded people younger than 16, people with dementia or learning disabilities, people receiving mental healthcare from a non-NHS (independent) mental health service and people who received care from more than one mental health trust during follow-up.

### Main outcome

Our main outcome was a readmission for acute mental healthcare, either to a psychiatric in-patient ward or for an episode of CRT home treatment. This outcome is a marker of relapse requiring treatment by acute mental health services; the same outcome was used in another study within our research programme.^7^ We also assessed predictors of the subgroup who were readmitted to in-patient wards.

### Exposures

#### Availability of ADUs in trusts

We compared acute readmission risk between NHS provider trusts which did and did not have access to an ADU. Information regarding ADU care could not be derived directly from the MHSDS because information on these services was not routinely coded in the data-set; the MHSDS only includes limited coding for ‘non-core’ NHS community mental health services. We therefore used data from a national mapping exercise of ADUs^3^ which identified 14 of the 56 provider trusts as having access to ADUs. The mapping work took place during the time period corresponding to the MHSDS data we analysed (2013–2015).

#### Variables for individuals

We extracted data for relevant variables based on (a) previous literature regarding acute admissions^2,8–12^ and (b) the availability and reliability of information within the MHMDS. These variables included sex, age, marital status, ethnicity and the Index of Multiple Deprivation (IMD) for the local area. IMD scores for 2010 were linked to the individual's Lower Super Output Area (LSOA). LSOAs are geographical units covering an average of approximately 1500 people. IMD scores are derived from multiple indicators according to an established methodology published by the Office for National Statistics. These routine data, their methodology and component indicators include but extend beyond census data.^13^ IMD scores were categorised in quintiles.

In terms of clinical presentation, we were unable to include clinical diagnoses owing to the large amounts of missing data on primary psychiatric diagnoses in the MHMDS. However, we defined three groups of broad diagnostic categories from a routinely coded MHMDS variable known as clinical care clusters. In the NHS, clinicians allocate care clusters for individuals on their mental health caseload. We categorised people into three groups based on other work using this data-set,^14^ namely non-psychosis, psychosis and severe psychosis (see Supplementary Table 1 available at https://doi.org/10.1192/bjo.2021.961).

We calculated length of stay (LoS) of the index acute admission as the number of days from the admission date to the discharge date.

### Statistical analysis

To assess risk of readmission to acute care, we fitted a multilevel logistic regression model with random intercepts at trust level to account for clustering of participants within trusts. In a null multilevel model (which contained only the random intercept and no explanatory variables), we estimated the proportion of variance in readmission which could be attributed to the trust provider, expressed as a percentage by estimating the variance partition coefficient.^15^

Next, we assessed individual-level characteristics associated with readmission, including the following covariates in the fixed part of the model: age, sex, marital status, ethnicity, clinical care cluster, LoS (in quartiles) of the index admission, whether the index admission was a CRT episode or in-patient care, and IMD (in quintiles). We also included a dichotomous trust-level variable indicating whether a trust provided ADUs in the local acute crisis pathway.

Results were considered statistically significant at the 0.05 level. Statistical analyses were performed using Stata version 15.1 (StataCorp, College Station, TX).

### Missing data

We used multiple imputation with chained equation, based on the assumption that the data were missing at random.^16^ We imputed missing data for care cluster (25.7% missing observations), marital status (21.7% missing), ethnicity (11.1% missing) and IMD (1.8% missing) using the financial year, age, trust, readmission and sex. We generated ten imputed data-sets and combined regression coefficients’ estimates across these using Rubin's rules.^17^

### Ethics and consent

The London Bloomsbury Research Ethics Committee granted ethics approvals for the study (ref. 16/LO/2160). Enhanced ethics approvals were granted by the Confidentiality Advisory Group owing to the use of routine clinical data for which patients had not individually provided consent for use in research (ref. 17/CAG/0101).

## Results

### Participant characteristics and risk of readmission

**Fig. 1:**
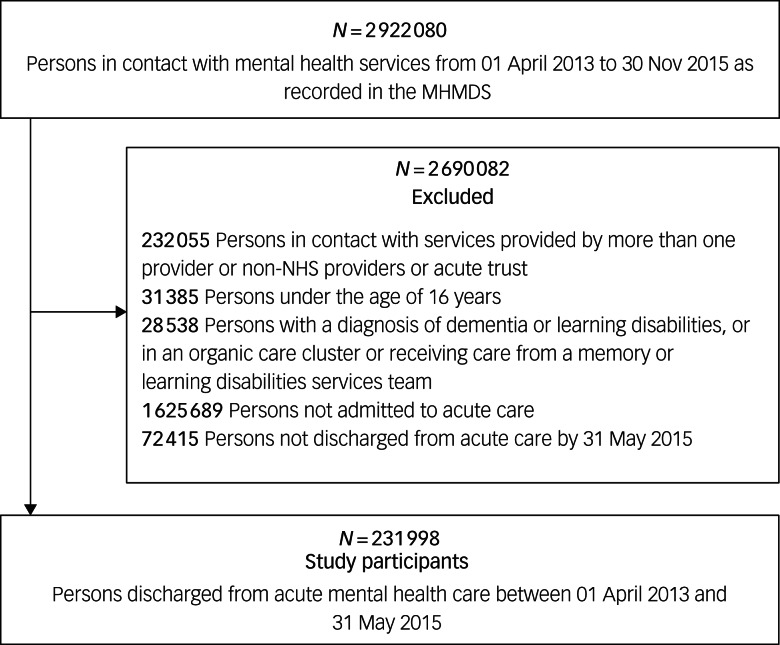
Flow chart describing selection of individuals discharged from an index episode of acute mental healthcare.

We identified eligible 231 998 individuals, in 56 NHS mental health provider trusts, who were discharged from the acute care pathway between April 2013 and May 2015 (Fig. 1). Median age at index admission was 40 years (interquartile range (IQR) 28–51), with approximately a 50% gender split. Among these individuals, 49 547 (21.4%) were readmitted within 6 months following discharge (Table 1), with a median time to readmission to acute care within the 6 months following discharge of 34 days (IQR 10–88).

**Table 1:** Characteristics of individuals discharged from acute care (index admission) in England between 1 April 2013 and 30 May 2015 and those readmitted to the acute care pathway or in-patient care only within 6 months after discharge.

	Discharged from index admission	Readmitted to the acute care pathway^a^	Readmitted to in-patient care
	*N* (%)	*N* (%)^b^	*N* (%)^b^
	213 998	49 547 (21.4%)	23 290 (10.0%)
Gender (*n* = 231 659)			
Male	115 075 (49.7)	23 961 (20.8)	11 580 (10.1)
Female	116 584 (50.3)	25 559 (21.9)	11 704 (10.0)
Marital status (*n* = 181 578)			
Single	109 181 (60.1)	26 417(24.2)	13 257 (12.1)
Married/civil partnership	44 126 (24.3)	9699 (22.0)	4665 (10.6)
Other	28 271 (15.6)	6752 (23.9)	3573 (12.6)
Age (*n* = 231 998)			
16–24 years	39 496 (17.0)	7665 (19.4)	2762 (7.0)
25–34 years	50 128 (21.6)	10 453 (20.9)	4790 (9.6)
35–44 years	49 170 (21.2)	10 705 (21.8)	4972 (10.1)
45–54 years	47 844 (20.6)	10 861(22.7)	5085 (10.6)
55–64 years	24 338 (10.5)	5625 (23.1)	2838 (11.7)
>64 years	21 022 (9.1)	4238 (20.2)	2843 (13.5)
Ethnic group (*n* = 206 309)			
White	174 983 (84.8)	39 571 (22.6)	18 690 (10.7)
Mixed	3487 (1.7)	865 (24.8)	486 (13.9)
Asian/Asian British	10 864 (5.3)	2227 (21.0)	1298 (12.0)
Black/Black British	10 245 (5.0)	2434 (23.8)	1634 (16.0)
Other	6730 (3.3)	1358 (20.2)	553 (8.2)
Clinical care cluster (*n* = 172 321)			
Non-psychosis	109 053 (63.3)	26 016 (23.9)	10 307 (9.5)
Psychosis	33 349 (19.4)	8921 (26.8)	5868 (17.6)
Severe psychosis	29 920 (17.4)	8988 (30.0)	6311 (21.1)
IMD (*n* = 227 872)			
1st quintile (least deprived)	23 415 (10.3)	4764 (20.4)	2244 (9.6)
2nd quintile	30 335 (13.3)	6291 (20.7)	2798 (9.2)
3rd quintile	40 543 (17.8)	8694 (21.4)	3949 (9.7)
4th quintile	55 871 (24.5)	12 360 (22.1)	5783 (10.4)
5th quintile (most deprived)	77 708 (34.1)	17 036 (21.9)	8349 (10.7)
Index admission			
Crisis team	156 968 (67.7)	32 446 (20.7)	16 310 (6.9)
In-patient ward	75 030 (32.3)	17 101 (22.8)	6980 (16.6)
NHS trust and ADUs			
Trusts with no ADU^c^	170 548 (73.5)	36 135 (21.2)	10 834 (9.6)
Trusts with ADU^d^	61 450 (26.5)	13 412 (21.8)	12 456 (11.4)
LoS in days, median (IQR)	11 (2−31)	10 (2−29)	14 (3−36)

IQR, interquartile range; IMD, Index of Multiple Deprivation; ADU, acute day unit; LoS, length of stay at index admission;

a. Includes in-patient care and crisis team care.

b. number of people readmitted as a percentage of the total number of people discharged.

c. 42 mental health trusts.

d. 14 mental health trusts.

**Fig. 2:**
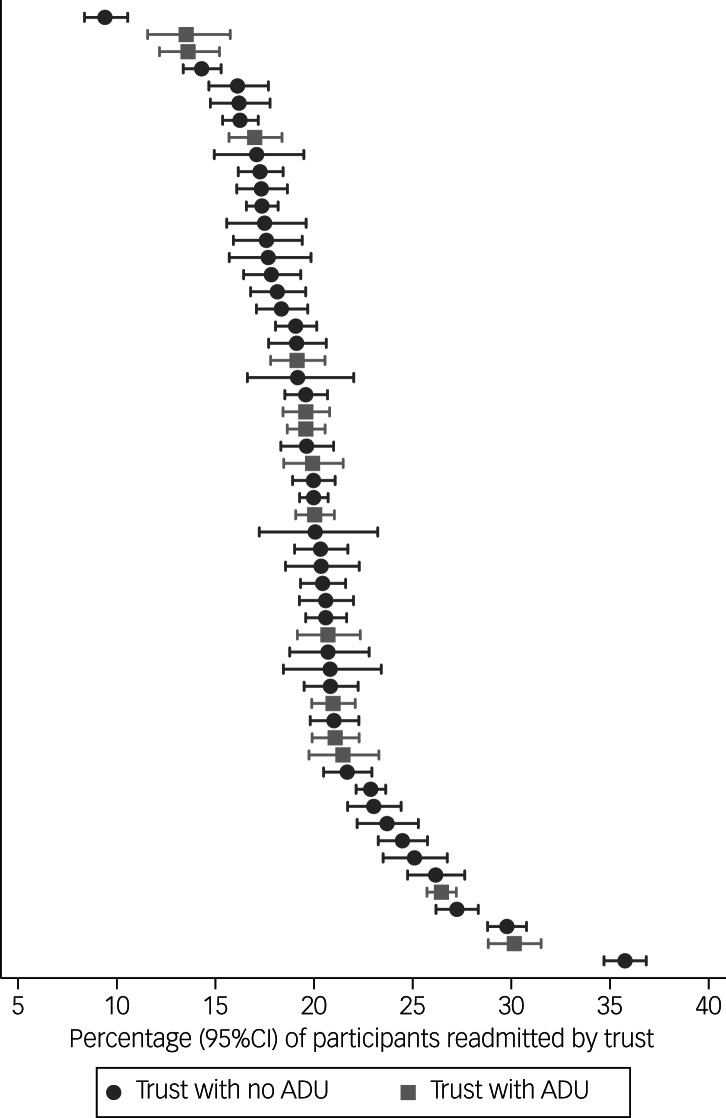
Crude percentage of participants who were readmitted to acute care treatment within 6 months by provider NHS trusts.

The clinical and sociodemographic characteristics of the participants are shown in Table 1. There was substantial variation in the proportion of participants readmitted within 6 months across different provider trusts (Fig. 2).

### Results of multilevel analyses

Results of the null model estimated that the overall odds of being readmitted across all provider trusts was 0.25 (95% CI 0.23–0.27). The variation partition coefficient showed that only 2.0% (95% CI 1.3–2.9%) of the variance in readmissions was attributable to provider trust-level factors. In other words, 98% of the variance was attributable to individual-level factors.

Results of the main multivariable analysis are presented in Table 2. We found no difference in risk in readmission between trusts with access to an ADU and those without an ADU (adjusted odds ratio (OR) 0.99, 95% CI 0.83–1.18). For individual-level variables, the following associations were found: belonging to older age groups, being female (compared with male), being single (compared with non-single), being of mixed ethnicity (compared with White ethnicity), being assigned a ‘clinical care cluster’ for psychosis (compared with non-psychosis), living in the most deprived areas (compared with living in the least deprived areas), being admitted to in-patient care (rather than CRT only) at the index admission, and shorter stay at the index admission.

**Table 2:** Predictors of being readmitted within 6 months after discharge from acute mental healthcare

	Multiple imputation model			
	Unadjusted OR (95% CI)	*P*-value	Adjusted^a^ OR (95% CI)	*P*-value
Age				
16–24 years	1.00		1.00	
25–34 years	1.11 (1.08, 1.15)	<0.001	1.09 (1.05, 1.13)	<0.001
35–44 years	1.18 (1.14, 1.22)	<0.001	1.14 (1.10, 1.18)	<0.001
45–54 years	1.24 (1.20, 1.28)	<0.001	1.20 (1.16, 1.25)	<0.001
55–64 years	1.27 (1.22, 1.32)	<0.001	1.23 (1.17, 1.28)	<0.001
>64 years	1.10 (1.05, 1.14)	<0.001	1.05 (1.00, 1.11)	0.041
Gender				
Male	1.00		1.00	
Female	1.07 (1.05, 1.09)	<0.001	1.14 (1.11, 1.16)	<0.001
Marital status				
Single	1.00		1.00	
Married/civil partnership	0.89 (0.86, 0.91)	<0.001	0.88 (0.85, 0.91)	<0.001
Other	1.01 (0.98, 1.04)	0.507	0.96 (0.93, 0.99)	<0.001
Ethnic group				
White	1.00		1.00	
Mixed	1.17 (1.08, 1.26)	<0.001	1.10 (1.02, 1.19)	0.019
Asian/Asian British	0.95 (0.90, 1.00)	0.028	0.91 (0.87, 0.96)	<0.001
Black/Black British	1.14 (1.08, 1.19)	<0.001	0.98 (0.93, 1.03)	0.352
Other	0.86 (0.81, 0.92)	<0.001	0.87 (0.81, 0.92)	<0.001
IMD				
1st quintile	1.00		1.00	
2nd quintile	1.02 (0.97, 1.06)	0.422	1.00 (0.96, 1.05)	0.864
3rd quintile	1.06 (1.02, 1.10)	0.006	1.03 (0.99, 1.08)	0.135
4th quintile	1.12 (1.08, 1.16)	<0.001	1.07 (1.03, 1.12)	0.001
5th quintile	1.15 (1.10, 1.19)	<0.001	1.08 (1.04, 1.13)	<0.001
Clinical care cluster				
Non-psychosis	1.00		1.00	
Psychosis	1.22 (1.18, 1.25)	<0.001	1.21 (1.18, 1.25)	<0.001
Severe psychosis	1.83 (1.78, 1.89)	<0.001	1.76 (1.71, 1.82)	<0.001
Index admission				
CRT	1.00		1.00	
In-patient ward	1.32 (1.29, 1.36)	<0.001	1.35 (1.31, 1.38)	<0.001
LoS at index admission				
<3 days	1.00		1.00	
3–12 days	1.09 (1.06, 1.12)	<0.001	1.01 (0.98, 1.04)	0.661
13–31 days	1.03 (1.00, 1.06)	0.052	0.89 (0.87, 0.92)	<0.001
>31 days	0.99 (0.96, 1.02)	0.440	0.74 (0.72, 0.77)	<0.001
NHS trust				
Trusts with no ADU	1.00		1.00	
Trusts with ADU	1.00 (0.86, 1.17)	0.985	0.96 (0.80, 1.14)	0.617

OR, odds ratio; IMD, Index of Multiple Deprivation (1st quintile, least deprived; 5th quintile, most deprived); LoS, length of stay for index admission; ADU, acute day unit.

a. OR mutually adjusted for all predictors.

Among the 49 547 individuals readmitted to acute mental healthcare, 47% (23 290) were readmitted to an in-patient psychiatric ward. The results of subgroup analysis suggested that people with psychosis of older age (>64 years) and of Black or mixed ethnicity were particularly at risk of being admitted to an in-patient ward within 6 months following discharge from acute care. The full results of this multivariable analysis of in-patient readmissions are presented in Table 3.

**Table 3:** Predictors of being admitted to in-patient care within 6 months after discharge from acute mental healthcare

Multiple imputation model				
	Unadjusted OR (95%CI)	*P*-value	Adjusted^a^ OR (95%CI)	*P*-value
Age, years				
16–24	1.00		1.00	
25–34	1.36 (1.29, 1.42)	<0.001	1.22 (1.16, 1.28)	<0.001
35–44	1.45 (1.38, 1.52)	<0.001	1.26 (1.20, 1.32)	<0.001
45–54	1.53 (1.46, 1.60)	<0.001	1.34 (1.27, 1.41)	<0.001
55–64	1.70 (1.61, 1.80)	<0.001	1.48 (1.39, 1.58)	<0.001
>64	1.95 (1.84, 2.06)	<0.001	1.63 (1.52, 1.75)	<0.001
Gender				
Male	1.00		1.00	
Female	1.00 (0.98, 1.03)	0.725	1.13 (1.10, 1.16)	<0.001
Marital status				
Single	1.00		1.00	
Married/civil partnership	0.89 (0.86, 0.92)	<0.001	0.85 (0.82, 0.88)	<0.001
Other	1.08 (1.04, 1.13)	<0.001	0.94 (0.90, 0.98)	0.006
Ethnic group				
White	1.00		1.00	
Mixed	1.32 (1.20, 1.46)	<0.001	1.20 (1.09, 1.32)	<0.001
Asian/Asian British	1.06 (1.00, 1.13)	0.054	0.97 (0.90, 1.05)	0.286
Black/Black British	1.47 (1.39, 1.57)	<0.001	1.11 (1.05, 1.17)	0.001
Other	0.78 (0.71, 0.85)	<0.001	0.79 (0.72, 0.87)	<0.001
IMD				
1st quintile	1.00		1.00	
2nd quintile	0.98 (0.92, 1.04)	0.436	0.96 (0.91, 1.01)	0.213
3rd quintile	1.04 (0.99, 1.10)	0.145	1.01 (0.95, 1.07)	0.755
4th quintile	1.10 (1.05, 1.16)	<0.001	1.04 (0.98, 1.10)	0.176
5th quintile	1.14 (1.08, 1.20)	<0.001	1.05 (0.99, 1.11)	0.078
Clinical care cluster				
Non-psychosis	1.00		1.00	
Psychosis	1.89 (1.82, 1.95)	<0.001	1.72 (1.66, 1.78)	<0.001
Severe psychosis	3.11 (3.01, 3.23)	<0.001	2.62 (2.52, 2.72)	<0.001
Index admission				
CRT	1.00		1.00	
In-patient ward	2.46 (2.39, 2.54)	<0.001	2.28 (2.20, 2.36)	<0.001
LoS at index admission				
<3 days	1.00		1.00	
3–12 days	1.37 (1.31, 1.43)	<0.001	1.05 (1.01, 1.09)	0.029
13–31 days	1.36 (1.31, 1.42)	<0.001	0.88 (0.84, 0.92)	<0.001
>31 days	1.51 (1.45, 1.58)	<0.001	0.68 (0.65, 0.71)	<0.001
NHS trust				
Trusts with no ADU	1.00		1.00	
Trusts with ADU	1.22 (0.96, 1.56)	0.103	1.09 (0.92, 1.29)	0.315

IMD, Index of Multiple Deprivation (1st quintile, least deprived; 5th quintile, most deprived); LoS, length of stay for index admission; ADU, acute day unit.

a. OR mutually adjusted for all predictors.

## Discussion

### Summary

We enrolled a large cohort of 231 998 people discharged from NHS acute mental healthcare in England and found that over 20% were readmitted to mental health acute care within 6 months from discharge. Half of these readmissions were to an in-patient psychiatric unit. The average time to readmission was 34 days. The proportion of people readmitted for acute care varied substantially across NHS provider trusts; however, most of this variance was due to individual-level factors rather than provider trust-level factors. The availability of an ADU in the local area did not predict readmission to acute care. The data-set did not contain enough information to allow more detailed assessment of the outcomes of people who use ADUs. Readmission to acute care was more common among people who were older, female, assigned to psychosis clinical care clusters, had a shorter index admission or had previously been admitted to an in-patient ward. The largest associations with readmission were seen among the severe psychosis group and those who were in-patients at baseline. We observed ethnic disparities in the likelihood of being readmitted, including to in-patient units, with higher risk among those of Black or mixed ethnicity compared with the White ethnic group.

### Clinical implications

Our findings suggest that a substantial proportion of patients re-enter the acute care pathway very quickly after being discharged from acute care. This might indicate that their ‘crisis’ has not been fully resolved during their period of acute care and that they remain clinically vulnerable after discharge. This is supported by the fact that people with shorter index admissions were at higher risk of acute readmission. Our results also suggest that individuals, especially people with psychosis, may require more effective interventions post-crisis to decrease the chance of relapse. Candidate interventions include peer support, self-management and crisis planning.^1,18^ For people with severe mental illness, primary care support has also been shown to decrease acute admission rates.^19^ It is well established that people from Black and minority ethnic groups have different pathways through acute psychiatric services for a variety of reasons, which require culturally appropriate intervention;^20^ our analysis suggests that this is not happening and that inequalities in care for different ethnic groups need to be addressed, and urgently.

There was little variance explained at the provider trust level, suggesting that it is the characteristics of a trust's catchment area population that determines readmissions, rather than variation among healthcare systems. The observation that limited variation could be explained at the level of each provider trust was also made in a previous UK study of compulsory psychiatric admissions in the NHS.^21^

There were no differences between readmission rates between trusts with and without access to ADUs, which was the focus of our overall research programme. This might be explained by the relatively low numbers of people who access ADUs compared with the large numbers who receive in-patient and CRT acute care. Published research suggests that the number of people using an ADU in each mental health trust is only 186 (IQR 114–2000) individuals per year.^3^ Our data did not allow us to explore the effectiveness (or acceptability) of ADUs or other acute care models at an individual level using this administrative data-set, as these episodes of treatment are not coded explicitly.

### Strengths and limitations of this study

The key strength of our study is the size and comprehensive national coverage of the MHMDS. To our knowledge, this is the largest study of national readmission rates to mental health acute care including in-patient wards derived from a large representative sample across England.

Readmission rates in these national data were lower than those seen in a previous cohort study of inner-London crisis team patients; however, that study had a longer follow-up of 1 year compared with 6 months in our study.^2^ In general, the individual risk factors for acute readmission were similar in both studies, particularly for people with psychoses and older people.

Our study had several limitations. First, the data were not contemporary as the routine data received from NHS digital are historical, and cleaning and constructing the cohort year on year is a complex, lengthy process. The follow-up period ended in 2015. There have been several changes in the English mental healthcare system since that time, including decreases in the number of acute beds available and increased use of community treatment orders.^14,22^ The MHMDS has large amounts of missing clinical data regarding diagnoses, and because of the nature of the administrative data we could not include a range of individual variables which might explain people's mental health vulnerability to relapse after they were discharged, for example, social support, employment, housing, individual-level deprivation, and receipt of community care and support for mental health. We also had no information regarding the pharmacological treatments people were receiving.

We were confident that readmissions to NHS crisis team and in-patient psychiatric services were captured comprehensively, but we did not identify admissions to other mental health providers in the UK including independent and voluntary sector providers of in-patient care, crisis houses or crisis cafes.

### Implications for research

Our findings indicate the need to understand delivery of care in more detail following discharge from acute care, particularly for people with shorter admissions. Such research will need to focus in more detail on the type of support which can decrease readmission rates, including for those most at risk of acute readmission such as individuals with psychosis, those in certain ethnic groups and people from more deprived areas.

The phenomenon of frequent readmission, including readmission to hospital, is not new and has been described in the international mental health literature,^23,24^ where a substantial proportion of people are reported to have multiple admissions. This highlights the need for effective interventions to prevent relapse following discharge into the community.

Furthermore, our research was limited by the availability of variables within routine administrative data-sets; this underscores how valuable it would be to have more robust and standardised coding of electronic records in clinical settings, for instance, regarding diagnosis categories, types of community mental healthcare, and ideally the specific interventions received by individuals.

### Clinical implications

Around one in five people discharged from acute mental healthcare in the English NHS are readmitted back to acute care within 6 months, and the median time to readmission is only 34 days. There is marked variation in readmission risk across trusts, but the reasons for this variation appear to be explained by differences in individual participant factors, including area-level deprivation, gender, older age, ethnicity and having a clinical care cluster indicative of psychosis. A particular concern was that people from Black and mixed ethnic groups were more at risk of in-patient admission. There was no evidence that trusts with access to ADUs had lower readmission rates, but our data source was not suitable for assessing whether individuals benefit from ADUs in terms of future readmissions. The results indicate the need for effective interventions to support people after discharge from acute care and reduce the requirement for readmission.

## Data Availability

The data used in this study were obtained from the Health and Social Care Information Centre (www.hscic.gov.uk), now known as NHS Digital. The agreements in place with the NHS Data Access and Advisory Committee for use of these data do not permit further distribution or sharing. Requests for the relevant data-sets must be made directly to NHS Digital. Further information can be obtained from the corresponding author.

## Supplementary material

For supplementary material accompanying this paper visit http://dx.doi.org/10.1192/bjo.2021.961. click here to view supplementary material
