# Supplementary material for: Readmission after discharge from acute mental healthcare among 231 988 people in England: cohort study exploring predictors of readmission including availability of acute day units in local areas
Source: BJPsych Open. 2021 Jul 19;7(4):e136. doi: 10.1192/bjo.2021.961 (PMC8329766; doi:10.1192/bjo.2021.961)
Supplement: Supplementary file 1 [file S2056472421009613sup001.docx]

***Supplementary Table 1. Categories of clusters for different mental health profiles in the dataset***

|  | **MHMDS coding** |
| --- | --- |
| **Care cluster** |  |
| Non-psychosis | Codes/Care Clusters: 00 – 09 (Variance, Low Severity Common Mental Health Problems, Low Severity Common Mental Health Problems with Greater Need, Moderate Severity Non-Psychotic, Severe Non-Psychotic, Very Severe Non-Psychotic Disorders, Non-Psychotic Disorder of Over-Valued Ideas, High Disability Enduring Non-Psychotic Disorders, Non-Psychotic Chaotic and Challenging Disorders, Cluster Under Review) |
| Psychosis | Codes/Care Clusters: 10, 11, 14, 15 (First Episode Psychosis, Ongoing Recurrent Psychosis [Low Symptoms], Psychotic Crisis, Severe Psychotic Depression) |
| Severe psychosis | Codes/Care Clusters: 12, 13, 16, 17 (Ongoing or Recurrent Psychosis [High disability], Ongoing or Recurrent Psychosis [High Symptoms and Disability], Dual Diagnosis, Psychosis and Affective Disorder [Difficult to Engage]) |
